# Degenerative Cervical Myelopathy and the Aging Spine: Introduction to the Special Issue

**DOI:** 10.3390/jcm9082535

**Published:** 2020-08-06

**Authors:** Aria Nouri, Renato Gondar, Joseph S. Cheng, Mark R.N. Kotter, Enrico Tessitore

**Affiliations:** 1 Department of Neurosurgery, Geneva University Hospitals, 1205 Geneva, Switzerland; rjag20@gmail.com (R.G.); Enrico.Tessitore@hcuge.ch (E.T.); 2 Department of Neurosurgery, University of Cincinnati College of Medicine, Cincinnati, OH 45267, USA; chengj6@ucmail.uc.edu; 3 Division of Neurosurgery, University of Cambridge, Cambridge CB2 1TN, UK; mrk25@cam.ac.uk

**Keywords:** introduction, focus issue, spinal cord injury, compressive myelopathy, spondylosis

## Abstract

Degenerative Cervical Myelopathy (DCM) is the most common cause of spinal cord injury in the world, but despite this, there remains many areas of uncertainty regarding the management of the condition. This special issue was dedicated to presenting current research topics in DCM. Within this issue, 12 publications are presented, including an introductory narrative overview of DCM and 11 articles comprising 9 research papers and 2 systematic reviews focusing on different aspects, ranging from genetic factors to clinical assessments, imaging, sagittal balance, surgical treatment, and outcome prediction. These articles represented contributions from a diverse group of researchers coming from multiple countries, including Switzerland, Germany, Italy, United Kingdom, United States, South Korea, and Canada.

Degenerative Cervical Myelopathy (DCM) is becoming a growing public healthcare burden, attributable principally to an aging population. It represents a group of degenerative changes of the cervical spine that result in static and dynamic compression of the spinal cord, leading to subsequent chronic inflammatory and mechanical damage to neural tissue [1,2,3]. DCM represents the most common cause of spinal cord impairment in the developed world, leading not only to a decrease in the quality of life of those affected but also is increasingly recognized as a public healthcare and social burden [3,4]. Over the past few years, research on this topic has allowed for a better understanding of its pathological features, natural history, diagnosis, severity, associated conditions, treatment thresholds, and outcomes, collectively helping to provide a better understanding of the condition [1,5]. However, this research effort has also clearly demonstrated the ongoing knowledge gaps that exist and require further investigation [2].

The present Special Issue in the *Journal of Clinical Medicine* was specifically dedicated to presenting current research topics in DCM. Twelve articles were published, comprising 1 narrative review and 11 original articles. The narrative review focused on the past perspectives, present developments, and future directions of DCM and is intended to provide an overview of the current status of DCM [1]. The editors of the issue contributed to this introductory review and decided to limit each of the past, present, and future sections to three themes in an effort to stay focused on the most important topics. The remaining 11 articles included 9 research papers and 2 systematic reviews focusing on different aspects, ranging from genetic factors [6] to clinical assessments [7,8], imaging [9,10], sagittal balance [11], surgical treatment [12], and outcome prediction [13,14,15,16] (Table 1). These articles represented contributions from a diverse group of researchers coming from multiple countries, including Switzerland, Germany, Italy, United Kingdom, United States, South Korea, and Canada. 

Several interesting findings were observed from this collective body of work. Starting with genetics, Pope et al. [6] evaluated the role of single genes in DCM onset, clinical features, and response to intervention and found genes with an effect on the radiological and clinical onset of spinal cord disease, correlating with the radiological and clinical severity of DCM. Polymorphisms of six genes were also found to have an effect on clinical response to surgery in DCM. The possible implications of this research are large, but further research will certainly be needed before this can be adapted from a clinical point of view.

Imaging-oriented research from D’Avanzo et al. [9] provided evidence that fractionated anisotropy (FA) values from MRI-DTI studies increase after decompression and potentially correlate with hand coordination and dexterity improvement, confirming previous reports that FA has an important role in prognostication. Fontanella et al. [10] reviewed the radiological finding of “snake-eye” appearance in the literature, finding some, albeit little, evidence supporting this appearance as a negative predictor. In their three illustrative cases, patients appeared to have relatively good outcomes, suggesting that further research is necessary to establish the clinical relevance of the “snake-eye” appearance. Kim et al. [11] tested the still complex and not fully understood concept of cervical sagittal balance and discussed the impact of cervical alignment on surgical decision making for laminoplasty. They concluded that the lack of kyphosis reducibility in cervical extension preoperatively should be considered as a contraindication to laminoplasty surgery.

Kalsi-Ryan et al. [7] focused on gait dysfunction in DCM, and demonstrated that DCM severity can be approximated using spatiotemporal gait parameters, providing another element of assessment that can be used to evaluate the degree and presence of functional impairment of patients. This assessment has the potential to also be used as an outcome predictor in future studies.

The majority of research focused on outcome prediction and prognostic factors. Severino et al. [13] supported the findings from D’Avanzo et al. [9], showing that FA can be used to predict surgical outcome and that increasing FA values preoperatively and postoperatively related with neurological function. Nouri at al. [14] investigated the relationship between gastrointestinal comorbidities (GICs) and DCM, as patients with GICs may suffer from anemia, inflammatory changes, and vitamin deficiencies which could be impact neurological healing. It was interesting to note that patients with and without GICs were not considerably different from a neurological function perspective, however, patients with GICs presented with a unique set of diverging characteristics including that they were more commonly female, and nearly a third of patients suffered from psychiatric comorbidities. Gembruch et al. [15] and Wilson et al. [16] focused on surgical outcomes for older patients and collectively showed that older patient clearly benefit from surgery, but may benefit less due to worse baseline neurologic function. Janssen et al. [12] reported a group of DCM patients in the context of rheumatoid arthritis, finding from their limited series that patients experience meaningful improvements in neurological function. They also showed that these patients are principally approached from a posterior approach initially.

Finally, in a topic relevant not only to the cervical spine, Panchagnula et al. [8] assessed the capacity of PROMIS to be used as a surrogate for EQ-5D, demonstrating the possibility of high accuracy mathematical transformations from PROMIS to EQ-5D in large cohorts, but limitations of accuracy of such transformation on an individual basis. This validation permits the use of PROMIS (a free questionnaire available from the NIH for the evaluation of quality of life) data to be used for quality-adjusted life year calculations.

It was the intention of this Special Issue to address a wide range of topics and we believe that the articles contained in the issue have largely achieved this objective. The editorial board also pursued this project with the hope of contributing new research to help tackle this increasingly prevalent and disabling clinical disorder. We would like to thank the various authors and peer-reviewers for helping to amass this excellent body of work.

## Figures and Tables

**Table 1:** Summary of published papers in this Special Issue.

Authors	Purpose	Study Design	Main Results	Conclusions
D’Avanzo et al. [9]	To evaluate the use of quantitative DTI in clinical practice as a possible measure to assess clinical outcome using the mJOA and hand dexterity.	Prospective observational	FA values increase after surgery, in particular, below the most compressed level (*p* = 0.044). Postoperative FA values tend to correlate with hand dexterity (r = 0.4272, R^2^ = 0.0735, *p* = 0.19 for the right hand; r = 0.2087, R^2^ = 0.2265, *p* = 0.53 for the left hand), but this relationship did not show statistically significance.	FA parameters on DTI, particularly below the site of compression, may be used as a marker of myelopathy. FA increases after decompression.
Pope et al. [6]	To evaluate the role of single genes in DCM, its onset, clinical phenotype, and response to surgical intervention.	Systematic review and meta-analysis	22 genes were found to have an effect on the radiological onset of spinal column disease, while 12 influenced clinical onset of spinal cord disease. Polymorphisms of eight genes correlated with radiological severity of DCM, while three genes had an effect on clinical severity. Polymorphisms of six genes were found to have an effect on clinical response to surgery in spinal cord disease.	There are clear genetic effects on the development of spinal pathology, the central nervous system (CNS) response to bony pathology, the severity of both bony and cord pathology, and the subsequent response to surgical intervention.
Nouri et al. [1]	Provide an overview of the history of DCM (notably the transition from cervical spondylotic myelopathy to DCM), discuss current developments and interesting future directions.	Narrative review	DCM causes neurological dysfunction and is a significant cause of disability in the elderly. DCM is triggered by a variety of degenerative changes in the neck, leading to alterations in alignment, mobility, and stability, and consequently, spinal cord compression. It is a growing health problem with recently published guidelines. Many studies are currently undergoing to better direct clinical management and improve treatment outcomes.	Significant progress has been made in the field, particularly in recent years, and there are exciting possibilities for further advancements of patient care.
Panchagnula et al. [8]	To compare six health score models in a cohort of adult spine patients and to assess their ability to map PROMIS-GHS to EQ-5D in the spinal population.	Validation, prospective questionnaire	Subgroup analysis showed good predictions of the mean EQ-5D by gender, age groups, education levels, etc.	The transformation from PROMIS-GHS to EQ-5D had a high accuracy of mean estimate on a group level, but not at the individual level.
Wilson et al. [16]	To evaluate the effect of older age on the functional and QOL outcomes after surgical treatment for DCM.	Ambispective, propensity-matched analysis. International, multi-center cohort.	Significant functional improvement from the baseline was greater in the younger cohort (1-mJOA 3.8 (3.2–4.4) vs. 2.6 (2.0–3.3) *p* = 0.007; 2-SF-36 physical component summary (PCS) and mental component summary (MCS) *p* ≤ 0.001, *p* = 0.007). Adverse events were not statistically significantly higher in the elderly cohort (22.4% vs. 15%; *p* = 0.161).	Elderly patients showed an improvement in functional and QOL outcomes after surgery for DCM, but the magnitude of improvement was less when compared to the matched younger adult cohort. An age over 70 was not associated with an increased risk of adverse events.
Kim et al. [11]	To examine whether cervical alignment influences surgical outcomes.	Retrospective	Patients with a cervical lordosis had an increase in upper cervical motion (C0-2 Range of Motion (ROM), C0-2ROM/C0-7ROM) after surgery, while the non-lordosis group exhibited a decrease in C2-7ROM and C0-7ROM. Lordosis was reduced in 12 patients (22%) after surgery. All six patients belonging to the non-reducible non-lordosis group (*N* = 6) before surgery remained in the same group after the surgery.	Cervical alignment and reducibility should be identified before surgery but do not correlate with spinopelvic parameters. Lack of kyphosis reducibility in cervical extension preoperatively is a relative contraindication to laminoplasty.
Gembruch et al. [15]	To determine the surgical benefit for older (>70 years) DCM patients.	Retrospective	Preoperative and postoperative mJOA were significantly lower in patients >70 years (*p* < 0.0001). Mean mJOA improvement did not differ significantly (*p* = 0.81) six months after surgery (G1: 1.99 ± 1.04, G2: 2.01 ± 1.04, G: 2.00 ± 0.91). The delay (weeks) between symptom onset and surgery (*p* = 0.003) and the duration of the hospital stay were longer for patients >70 years old (*p* < 0.0001).	Preoperative and postoperative mJOA are affected by the patients’ age, but improvement is similar. Patients should be considered for DCM surgery, regardless of their age.
Nouri et al. [14]	To investigate the difference between patients with or without gastrointestinal comorbidities (GICs) who are surgically treated for DCM.	Ambispective. International, multi-center cohort.	GICs were present in 121 patients (16%). These patients were slightly less neurologically impaired based on the Nurick grade (3.05 ± 1.10 vs. 3.28 ± 1.16, *p* = 0.044) and had a worse physical health score (32.80 ± 8.79 vs. 34.65 ± 9.38 *p* = 0.049), worse neck disability (46.31 ± 20.04 vs. 38.23 ± 20.44, *p* < 0.001), a lower prevalence of upper motor neuron signs (hyperreflexia, 70.2% vs. 78.9%, *p* = 0.037; Babinski’s sign 24.8% vs. 37.3%, *p* = 0.008), and a higher rate of psychiatric comorbidities (31.4% vs. 10.4%, *p* < 0.0001). On MRI, GIC patients less commonly exhibited signal intensity changes (T2 hyperintensity, 49.2% vs. 75.6%, *p* < 0.001; T1 hypointensity, 9.7% vs. 21.1%, *p* = 0.036), and had a lower number of T2 hyperintensity levels (0.82 ± 0.98 vs. 1.3 ± 1.11, *p* = 0.001). There was no difference in surgical outcome between the groups.	DCM patients with GICs are more likely to be female and have significantly more general health impairment and neck disability, and more commonly exhibit psychiatric comorbidities. However, these patients have less clinical and MRI features typical of more severe neurological impairment.
Kalsi-Ryan et al. [7]	To test if spatiotemporal gait parameters, including the enhanced gait variability index (eGVI), could be used to sensitively discriminate between different severities of DCM.	Prospective observational, cross-sectional	A significant correlation was found between the mJOA score and eGVI. Significant differences in the eGVI (X^2^(2, *N* = 153) = 55.04, *p* < 0.0001, ε2 = 0.36) were found between all groups of DCM severity, with a significant increase in the eGVI as DCM progressed from mild to moderate.	The eGVI was the most discriminative gait parameter and correlated with the severity of DCM. Quantitative gait assessments are an objective tool to diagnose, classify, and evaluate the impact of therapeutic interventions in DCM.
Severino et al. [13]	To evaluate the capacity of conventional and advanced MRI techniques (using DTI), and neurophysiological parameters to identify the best candidates for decompressive surgery.	Prospective observational	There were no statistical differences in age, T2 hyperintensity, and midsagittal diameter between best and normal responders. There was a significant inverse correlation between the MEPs central conduction time and mJOA in the preoperative period (*p* = 0.0004), and a positive correlation between fractional anisotropy (FA) and mJOA during all the phases of the study, and statistically significant at 1-year (r = 0.66, *p* = 0.0005). FA was significantly higher amongst “best responders” compared to “normal responders” preoperatively and at 1-year (*p* = 0.02 and *p* = 0.009).	FA and electrophysiological aspects have a role in the diagnostic a prognostic evaluation of DCM. These results support the concept of a multidisciplinary approach in the assessment and management of DCM.
Janssen et al. [12]	To describe the rare but important presentation of cervical myelopathy in patients with rheumatoid arthritis, and its management.	Retrospective study and narrative review of literature	All patients received surgical treatment via posterior fixation, and in addition, two of these cases were combined with a transnasal anterior approach. mJOA improved from 12 ± 2.4 to 14.6 ± 1.89 at a mean follow-up at 18.8 ± 23.3 months (range 3–60 months) in five patients.	Posterior approaches are preferred for craniocervical junction instability and DCM in the context of rheumatoid arthritis. Fixation in addition to cord decompression is generally required.
Fontanella et al. [10]	To discuss the role of snake-eye appearance on MRI and its relationship with prognosis.	Case series and systematic review of the literature	Three studies which discussed snake myelopathy were reported comprising a cohort of 144 patients. “Snake-eye” appearance was regarded as a negative prognostic factor in particular, in Mizuno’s study, the improvement ratio determined by JOA score was 32.2% in SEA (snake-eye appearance) vs. 47.1% in non-SEA, and 50% (*p* < 0.01) in control cases, in which high signal intensity was absent.	“Snake-eye” myelopathy represents a rare form of myelopathy and the pathophysiology is still unclear. The frequency of this presentation may be greater than previously thought and appears to be a negative prognostic factor.
